# Selections and Abstracts

**Published:** 1901-11

**Authors:** 


### SELECTIONS AND ABSTRACTS.

### Twentieth Century Surgery.

The executive committee has requested me to discuss the subject of "Twentieth Century Surgery," with the understanding that my remarks shall be neither too technical nor too scientific—that they shall be addressed to the laity rather than to the faculty. Hence, I trust that the physicians here will pardon me if I dwell upon subjects with which they are already sufficiently familiar.

Believing that things present are best appreciated by contrasting them with things past, I shall speak of ancient surgery before considering that of to-day. My address, however, being limited to twenty minutes, I shall confine myself largely to the subject of amputations, using this as an illustration of surgery as a whole.

Let us imagine an amputation during the tenth century. While half a dozen assistants sat upon the patient and subdued his wild struggles, a surgeon, with a great knife shaped like a sickle, cut straight down through the skin and muscles of an injured leg like slicing a sausage or a cucumber. There was no chloroform then. Chloroform is a modern luxury and was not discovered until the middle of the nineteenth century. A man in those days had to shut his eyes, dig his nails into the palms of his hands and bear the torture as best he could. An operating-room was truly a hell of anguish. No flaps of any kind were made in amputating—simply a great, gaping, bleeding wound, with the bone protruding from its center. And the bleeding was something awful, the blood spurting from severed vessels in drenching streams. Nothing was known of stopping the circulation by constriction of the limb with a tourniquet, and it was no uncommon thing to see a poor, shrieking wretch bleed to death upon the operating-table. What a memory that must have been to keep close to one during a dark night! No wonder surgeons were often afraid to operate.

As soon as the bone had been hastily divided by a saw (how a saw grates on a bone), a red-hot iron was rubbed again and again across the raw and quivering muscles—think of the agony of it—in order to sear the open vessels and stop the terrible hemorrhage; for the art of tying arteries with ligatures of thread did not come into general use until the sixteenth century.

Instead of using a hot iron, the stump was sometimes plunged into boiling oil, melted sulphur, liquid lead or boiling water. You could take your choice. The ancient Hindus covered the raw and bleeding part with molten pitch. Always give me an ancient Hindu for a cheerful idea.

Such was the fear of hemorrhage that many ingenious expedients were employed to control it. A favorite remedy among learned men was to hold a toad in the hand until the animal became warm. But toads have an unreasonable tendency to remain cold, so the advocates of this attractive method had always an excuse ready in case the patient failed to receive that benefit which he had a right to expect. It was claimed in support of this theory that if a toad were hung about the neck of a rooster for a day or two the head of that rooster could be cut off without the loss of a drop of blood. Roosters are different now.

Another method was to thrust the bleeding part into the vitals of a hen. But while the surgeon was out in the yard chasing a hen, most patients would bleed to death.

The moss that grows within dead men's skulls was a favorite, if somewhat gruesome, application. If they used the moss that grew upon their surgeon's backs, there would have been less trouble in obtaining it, and the results might have been just as good.

Various mysterious powders and liquids were employed, which were as efficacious as so much sand, but which were nevertheless bought for fabulous sums, occasianally by kings and princes. Some of these powders were so potent that they would even prevent hemorrhage when applied to the weapon which produced the wound. All the timid duelist had to do was to get a little powder secretly smeared upon his antagonist's sword, and the results after that might take care of themselves.

We cannot wonder that a reaction set in, many surgeons becoming afraid to amputate at all. Limbs were no longer cut off, but were permitted to rot off, during months of fever and suffering.

Other operators amputated with red-hot knives, while some past masters in the arts of torture used wooden or ivory knives dipped repeatedly in nitric acid.

The more quickly a leg was removed the sooner could bleeding be controlled, hence a pious man invented a machine resembling a guillotine, in which the limb was placed on the edge of a knife-blade, while a second knife, heavily weighted, fell upon it from a considerable height. Bones were, of course, terribly splintered, but no one seemed to pay much attention to a few splinters. What a delightful time the victim must have had waiting for that knife to fall.

Another eminent practitioner of the gentle art of surgery tied a cord about the seat of the amputation, and, by means of a stick, slowly twisted that cord tighter and tighter during the course of several abnormally long days, until the leg was twisted off. That surgeon is now shoveling sulphur in the warmest corner of purgatory.

Those who calmly chopped off legs with a meat-ax were not so cruel after all, considering the performances of their brothers in iniquity; but there is no excuse for the villains who used a chisel and mallet, cutting slowly through flesh and bone as though doing a job of carpentering.

As late as the middle of the present century a Parisian surgeon first fractured the bones of his patient's limbs and then twisted off the rest of the tissues with a wire rope. A pity such men must die. They should live forever and enrich the world with their valuable discoveries.

It is strange that surgery made so little progress for centuries—from the time of the Roman Empire to within two or three hundred years of the present date. Throughout these dark ages of medicine many surgeons feared to amputate through living tissues, but first permitted injured extremities to become foul and gangrenous, and then cut them off through the dead parts. This was the only procedure followed during the time of Hippocrates. Hemorrhage was avoided with certainty, even though the patient died.

Under these old methods it required from six to eighteen months to recover, during which dreary eternity the end of the bone, which always protruded from the open wound, had to exfoliate and drop off. As an aid to this process the bone was repeatedly burned with a red-hot iron. This kept the patient in good heart, and made him realize that something was being done. Even if the sufferer was unlucky enough to recover, which seldom happened, his stump was covered with a tender and delicate scar which was always ulcerating and giving rise to pain and annoyance.

When the tourniquet came into general use and the fear of hemorrhage was lost, amputation experienced a boom—everybody wanted to amputate and everybody amputated. A surgeon's repuation was largely measured by the number of mutilated cripples hobbling in his wake. If a man hurt his leg at all severely, that was the end of that leg. Amputations were made even for simple fractures. The craze, however, soon subsided, and the present rule became fairly established—to save whenever saving is possible.

Not so very many decades ago, to enter a hospital was almost equivalent to entering the jaws of death. Gangrene, erysipelas and blood poisoning stalked unhindered through the overcrowded and poorly ventilated wards. So prevalent became the most horrible and hopeless forms of wound-disease that the hospitals of Paris and Vienna were formally closed and patients housed in tents in the open fields. The fear of hospitals which still exists in the minds of many probably dates largely from this period.

Nothing was known of surgical cleanliness until some thirty-five years ago, when Lister, of London, announced his momentous discoveries. Suppuration was regarded as not only unavoidable, but desirable and even necessary. Surgeons were never satisfied until good "laudable" pus had been obtained; and the relations between fever, blood poisoning and suppuration were unrecognized or disregarded.

To have a leg amputated, even in the twentieth century, is not a luxury, nor a thing to be strongly desired; but it is not the horrible, blood-curdling experience now that it once was. Some wet morning, for instance, when you least expect it, you slip and fall in front of a trolley car on the corner of Seventeenth and Curtis. You find yourself on your back in the street, your leg crushed below the knee, skin the color of white wax, and clothes muddy and torn. After a wild ride in the ambulance, with swinging slides around the corners, and painful jolts over the car tracks, you reach the hospital. A hypodermic of morphine is given, and in a few minutes you are perfectly easy and free from pain, although weak, pale and bloody. A surgeon examines your affected extremity. The skin is burst open, exposing the lascerated muscles and splintered bones. The circulation is destroyed and the whole dreadful wound is smeared full of the dirt of the street. An amputation is necessary. Before you fully grasp the horror of your position, for the mind is hazy at such times, chloroform is administered. * * * And then you find yourself back in bed, gazing at a sweet young woman in a white cap and spotless apron, and trying to realize that your wounded leg is no longer attached to your body. When the effects of the anesthetic have worn off, you are quite comfortable, and in two or three weeks you are well and about on crutches. From the time you entered the hospital there was practically no pain, no fever, and comparatively little discomfort.

Some years ago 222 major amputations were reported from the Heidelberg Clinic, 200 of them progressing in this eminently satisfactory manner, and the results are even more brilliant to-day.

The achievements and possibilities of twentieth century surgery are so well known that I am tempted to rest my case here, for even the uninitiated must appreciate the contrast between the old and the new, without further elucidation.

Our present superiority, however, does not depend, as some believe, upon greater skill in the use of the knife, for our more recent ancestors could operate just as skilfully as we do, and some of them even outclassed us in dexterity. But the brilliant success of modern surgery rests upon improvements in hospitals and in nursing, upon surgical cleanliness and a knowledge of bacteriology, and upon advancement in methods of diagnosis with the resulting increase of "surgical judgment."

The fear of hospitals has largely disappeared, and people are so rapidly learning to regard them as havens of comfort and safety that the numerous institutions can scarcely find room for those who are constantly applying for admission. Cleanliness, neatness and cheerfulness reign where formerly existed dirt, contagion and despair.

The trained nurse has added much more to the comfort and safety of operative procedures than is perhaps generally appreciated. Our palatial operating rooms, with their plate glass and polished marble, would be but foolish pretense without the intelligent supervision of a well-trained nurse.

Diagnosis has advanced with gigantic strides within the last few years. To be able to recognize a disease with certainty, and to recognize it promptly, is of the utmost importance, perhaps involving the life of the patient. Microscopic examinations of the blood often enable us to detect deep-seated suppurations and to distinguish one disease from another; they may even tell us whether the individual is strong enough to bear an operation. From laboratory study of the bodily excretions and secretions the most pathological secrets of internal organs are exposed, and even the ignorant are familiar with the wonderful possibilities of the Roentgen rays. All accessible cavities of the body, even the stomach, may be explored by means of mirrors and electric lights, and operations performed within them which could scarcely have been imagined by older surgeons. Experience has taught us that various diseased portions of the body can be removed with benefit—not only arms and legs, but kidneys, bladders, spleens and even stomachs. It is no uncommon thing for surgeons to take out a section of intestine, a portion of the liver, or even a part of the lung or brain.

However important other things may seem, bacteriology is really the hub around which the spokes of twentieth century surgery revolve. Without the foundation of bacteriology and the knowledge of surgical cleanliness which it involves, the entire superstructure of modern surgery would tumble into fragments. Bacteriology is sometimes spoken of as a theory. It is not a theory. It is a science, the facts of which are as absolutely demonstrated as those of chemistry, when certain germs are invariably found in diseased wounds, when we can cultivate them on gelatine in pure cultures through many generations, and then inoculate them into animals and prodnce the same sort of inflammation containing the same germs—when we can do these things as often as we please, we are dealing with facts and not with theory—facts which are practically supported by brilliantly successful surgery.

The art of surgical cleanliness, which means the exclusion of germs from wounds, has added largely to the sum total of human happiness and longevity. Much physical suffering, which was formerly endured with what fortitude we could command, is now dissipated by a few painless strokes of the knife, and with a minimum amount of danger and discomfort. Especially should women be thankful for the blessings which have been conferred upon them.

The twentieth century has opened most propitiously as far as surgery is concerned, and the horizon is replete with suggestions of further discoveries and improvements. And one of the greatest advances will be an increase in our knowledge of what to do and what not to do—a clarification, as it were, of the surgical atmosphere.—*Leonard Freeman, M.D., in Denver Medical Times.*

---

### A CLINICAL REPORT ON GUDE'S PEPTO-MANGAN.*

There may still be some doubt whether manganese is a normal constant constituent of the human blood or of any of the tissues of the body. It may not have been positively determined whether iron, when given in an inorganic compound or in pure metallic form, is absorbed by the mucous membrane of the stomach or intestinal canal, or whether it accomplishes its curative work by some occult process of stimulation of that membrane, by virtue of which it takes up with greater readiness the nutritive portions of food substances which are presented to it at the same time; or whether it plays a chemical rôle in changing the contents of the alimentary canal, so that what eventually passes into the circulation is more fitted to maintain high standards of nutrition or will prove less deleterious to the processes of life.

Even when we have combinations which, whether obtained synthetically or analytically, resemble the forms in which this metal is found in the blood, our assurance is by no means perfect that

---

*By Samuel Wolfe, A.M., M.D., Physician to Philadelphia Hospital, Neurologist to Samaritan Hospital, Philadelphia, Pa.

they can pass the portals of the circulation, the absorbent organs of the alimentary tract, without great risk of change from their original forms, in their contact with the substances and tissues to which they are exposed.

All these are still questions on some of which the evidence is sufficiently positive to leave but little doubt, while on others there are so many theories that we are left to choose what may best suit the results of our own observation, if not, indeed, our caprice or fancy.

To the chemist and therapeutist these are certainly interesting and practical questions. Before the physiologist and pathologist still others of equal importance loom up. What are the different steps in the process by which an atom of iron, in either a food or drug, becomes ultimately an ingredient of the hemoglobin of a corpuscle, and what have been the dynamic processes by which it has associated itself up to this point? Again, what is its final destination and disposal? With what materials has it been combined, and what forces has it generated and modified by the time it has finished its course? What accounts for its disappearance under certain abnormal conditions, and why does the train of symptoms which we witness arise under these circumstances?

Again, these are facts, theories, hypotheses and speculations which we are bound to consider, and, in the light of our own reason and judgment, to determine.

But while we are thankful for all the light that can be shed on these problems, and, as members of a cultured profession, are impelled to continue their investigation, yet to the clinician their solution is not essential. Whether his path be flooded with the brightness of midday or shrouded in Egyptian darkness, he must still walk on in it. When, in the records of professional literature or in the acquirements of his own personal experience, certain means have associated themselves with consequent legitimate ends, it is his plain duty to adapt the one to the other. And, again, where the means have been to a degree inadequate, on the introduction of what appeals to his reason as of a higher probable power, he must determine the claim. The clinician must not allow himself to be diverted too far into the by-paths of knowledge, lest he become timorous and undecided. The locomotive engineer, who knows the management of his engine in such a way as to start it, regulate its speed, and stop it, so that he will constantly carry his train to its destination on time and without accident, and with the accomplishment of all that is expected of him at the termini and at the way-stations, is but little the better for a complete knowledge of the country through which he travels; of the industries of the towns at which he stops; of the mechanical and physical forces which rule the movements of his engine; or of the mathematical rules which govern the construction of the road.

My observations with Pepto-Mangan, introduced to the profession by Dr. Gude, chemist, of Leipzig, are such as can be easily confirmed by any physician, since they were all made in private practice, and rest on bedside and office notes. I have used the preparation to a considerable extent ever since it was first brought to my notice, which I think was about two years ago. Owing to some specially good results obtained, I was led to the series of recorded observations on which this paper is based. They extend over four months of time, and embrace about fifty cases.

As a rule, I followed the directions issued by the manufacturers in its administration, giving to an adult a tablespoonful dose and to younger subjects a proportionate amount. Milk seemed to be the best vehicle, and immediately before or after meals a convenient time. In its relation to food, however, I do not think we need exercise any special care as to its administration. There were but few cases in which I found any disturbance of the digestive functions by these doses, but in several there was considerable constipation induced, and in one or two some diarrhea as the apparent result of the drug. While my experiments in this direction have not gone far enough to beget firm convictions, I am of the opinion that in the main equally good results could be achieved by a smaller average dose, and in this way the small number of untoward results might probably be still further diminished.

In one series of twenty-three cases the patients were all married women, ranging from the ages of twenty-two to seventy, who were more or less anæmic from various causes. In all but five the results were decidedly satisfactory, and of these one failed to report the second time, so that the result is not known. The other four were cases of advanced organic disease, in which no therapeutic procedure could have given decided results. In nine of the twenty-three cases the results might be classed as brilliant. In all of the others I am convinced that no other preparation of iron could have done more. The condensed details of a few illustrative cases from this series follow.

A woman of sixty-five, during several years, had occasionally applied for relief from vertigo, frequent attacks of palpitation and general weakness and nervousness. She also had frequent long-continued attacks of diarrhea and some gouty manifestations in the joints. In November I found her very decidedly prostrated and anemic. She took the Pepto-Mangan in connection with a carefully regulated diet (chiefly albuminous) for six weeks, and gained steadily in strength and weight. At the end of that time her symptoms had disappeared, and she claimed to be in better condition than at any time during the previous two years.

A woman of twenty-five, of highly nervous temperament, cultured and refined, had passed through her first confinement in May, the labor being a very difficult one, and resulting in a still-birth. She grieved very much, and, though fighting very bravely against her depression of spirits, by autumn she became very neurasthenic and anemic. She had morbid fears, frequent flushes, and some menorrhagia. She was put to bed and given Pepto-Mangan and strychnia sulphat in gr. 1-30 doses b. i. d., and recovered rapidly. She again became pregnant, and is perfectly well.

A mother of three children, aged thirty-two, the youngest ten years of age, who has during the last year had some three or four attacks of menorrhagia, had gradually reached a quite profound state of anemia in spite of plentiful administration of other forms of iron in the intervals of the menses. She is obstinately persistent in refusing a uterine examination, and was therefore treated symptomatically only. My recent prescription of Pepto-Mangan has rapidly dissipated her pallor and improved her general health.

A primipara, aged twenty-two, was pale during pregnancy, and at the end of her lying-in, though she had not lost blood at all profusely, and claimed to feel well, was very pallid. After using Pepto-Mangan for two weeks her color had been fully restored.

Two young married women, both of whom had passed through a confinement within a year, were anemic, and frequent sufferers from headaches, and considerably debilitated. They both recovered promptly on the Pepto-Mangan.

Another series of nine cases consists of children from infancy to the age of twelve. In all marked results were obtained.

A little girl of four, for two successive summers had frequent malarial attacks of an irregular character and resulting in anemia and debility. She had been treated with arsenic, quinine, various preparations of iron, and, though responding to the drugs, was still inclined to fall always a victim to fresh onsets of the disease. On Pepto-Mangan she made steady and rapid progress toward robust health, and now is a perfect specimen of a vigorous child.

An infant of seven months passed through a siege of infantile remittent with a great deal of bowel disturbance, which yielded to quinine in the course of two weeks. Within a month the same train of symptoms developed, and quinine was again given, and followed by Pepto-Mangan, and since then the child's health has remained good, although several months have elapsed.

A girl of seven, who had for a long time been pale, took diphtheria. After recovery from the disease, the anemia, as might be expected, was still more grave. She was put on Pepto-Mangan and soon became rosy and strong.

Another girl of the same age, also habitually pallid, had wry-neck for two weeks, which disappeared under iodide of potassium, but the anemia had increased. Her restoration in color and to robust health was secured by the use of Pepto-Mangan for a month.

A little boy of four had measles, from which he made a good recovery. Two months later he was very anemic and listless, with poor appetite and slight feverishness. He at once improved on the Pepto-Mangan, and continued until fully restored.

A baby, six months old, one of a pair of twins, had developed quite a marked degree of hydrocephalus. Large thin blue veins stood in relief all over the scalp. The anemia was very pronounced. She was put on Pepto-Mangan, and her appearance now is much better, with strong indications of the arrest of progress in the disease.

Another series of five cases includes girls approaching or slightly beyond puberty, all anemic, and all responding to the use of Pepto-Mangan.

Of this class, a girl of seventeen, who has always been pale, thin and puny, has only come under treatment within a month. She has never menstruated, and shows but little tendency to don the usual habiliments of the maiden. She is under size, but has, since her early girlhood, always had an aged look. Her appetite is very meager and somewhat capricious. She suffers from pains in the legs, more especially the joints, and has a distinct systolic murmur. Under the Pepto-Mangan she seems to gain in color and appetite, and the pains in the legs have somewhat diminished. I shall watch the outcome of this case with great interest.

In submitting this report I wish to summarize these conclusions:

That Pepto-Mangan is a highly available preparation of iron, on account of its liquid form, pleasant taste, non-corrosive action on the teeth and unirritating effect on the digestive organs, admitting thus of easy gradation of dose, easy administration to children and avoidance of unpleasant effects in all classes of patients.

That it is an efficient and rapid restorer of the normal quality and quantity of the blood, in all conditions where the state of the organism admits of this result by the administration of a chalybeate.

---

### A New Continued Fever.

E. J. Spratling, of Georgia, in a communication to the *Medical Record* of September 7, 1901, has the following to say:

"We had here in the spring and early summer what is to me a new fever. The trouble began with malaise for three or four days, then muscular pains, especially of the neck; there were slight chilly sensations; the temperature rose to from 100° to 102°, the pulse was 110 to 120, the respiration 30 to 40; there was nausea, the patient had a hacking cough and mental depression, sometimes amounting to melancholia. The following day the pains were more pronounced, especially in the long bones and joints; headache was severe and accompanied with photophobia and dizziness on rising; the temperature was 104 to 106°, pulse 120 to 140, respiration 40 to 60 and shallow; the abdomen was sore and tympanitic; there was gastralgia, with disgust for food; the urine was scanty, offensive and highly colored. The mind was periodically clouded, often actively delirious, according to the temperature, which changed rapidly, sometimes four or five marked fluctuations in the twenty-four hours; the slightest noise, disturbance, or exertion, mental or physical, caused rapid exacerbations. This was one of the characteristics of the disease.

"Thus far this fever closely resembled dengue, and might in a few cases be confounded with grippe. But in dengue the cases of a given epidemic will be much alike as to violence; in this the widest possible range was found. In several cases the temperature reached 106.5°, in many not 100°. In dengue the surroundings have but little influence; in this they were nearly all-powerful. In dengue whole families are usually affected; in this rarely more than one member. The temperature of this fever was very susceptible to the action of antipyretics.

"The symptoms given above lasted from two to five days, then gave way rapidly, all discomfort disappearing; the appetite returned, the pulse fell to from 85 to 100, the respirations 30 or 40, and the patients expressed themselves as 'feeling fine; could get up if only stronger.' But the erratic fever and scant urine persisted for from three to ten days longer, and there were frequent profuse sweats. Then the fever ceased and the kidneys acted normally; the mind became clear, but mental and physical lassitude persisted in some cases for weeks.

"Thus we see we have a close congener of, if not actually, Malta or Mediterranean fever.

"Out of about sixty cases we found nearly one-half slight, confining the patient to bed only two or three days—the longest period was twenty-three days. The complications, in order of frequency, were bronchitis, enteritis, laryngitis, pneumonia, pleurisy, arthritis, and lymphangitis. The prognosis was good when no organic heart lesion existed, when the strength was preserved, quiet was maintained, the kidneys were stimulated with alkaline diuretics, and depressant medication was avoided.

"This is a high, dry locality, and neither malaria nor typhoid fever exists here; nor did any tests for either of these diseases give positive results in this fever."

---

### SCIENTIFIC STUDY OF THE CRIMINAL AND DEFECTIVE CLASSES.

An effort is being made to establish a laboratory in the Department of the Interior at Washington for the practical application of physiological psychology to sociological and abnormal or pathological data, especially as found in institutions for the criminal, pauper and defective classes and in hospitals, and also as may be observed in schools and other institutions. The defect in our present criminal law is, as we have before remarked, that it regards the crime and not the criminal. It presupposes that all mankind possess an equal power of resistance to anti-social tendencies. It practically lays down as an axiom that the child born of criminal parents, brought up in an environment of crime, is, until he has actually come within the jurisdiction of a magistrate's court, as equally desirable a citizen to all intents and purposes as he who has been reared in the atmosphere of the law-abiding.

Until an offense has been committed the law does not recognize the offender. For it the prospective criminal does not exist. Unfortunately there are some beings who are moral imbeciles. To confine our efforts to punishing crime when committed rather than to preventing its commission is like the proverbial locking of barn after stealing of horse. Nothing has been done by government as yet to treat the matter scientifically, and when it is considered that $600,000,000 is the annual tribute which, statisticians assure us, society pays to crime, and that the United States has the highest murder rate of any civilized country in the world, one is almost tempted to long for a return to the condition of things when one hundred and sixty offenses were punishable by death, though it be conceded that the death-penalty is one of the slightest of deterrents to crime. The promoters of the measure have our best wishes. As put by the well-known writer . . . . "The study of man, to be of most utility, must be directed first to the causes of crime, pauperism, alcoholism and other forms of abnormality. To do this the individuals themselves must be studied. As the seeds of evil are usually sown in childhood and youth, it is here that all investigation should commence, for there is little hope of making the world better if we do not seek the causes of social evils at their beginnings."—*Editorial in "The American Lawyer," New York.*

---

### NICHOLAS SENN PRIZE MEDAL.

The committee on the Senn Medal beg leave to call attention to the following conditions governing the competition for this medal for 1902:

1. A gold medal of suitable design is to be conferred upon the member of the American Medical Association who shall present the best essay upon some surgical subject.

2. This medal will be known as the Nicholas Senn Prize Medal.

3. The award will be made under the following conditions: *a.* The name of the author of each competing essay shall be enclosed in a sealed envelope bearing a suitable motto or device, the essay itself bearing the same motto or device. The title of the successful essay and the motto or device is to be read at the meeting at which the reward is made, and the corresponding envelope to be then and there opened and the name of the successful author announced. *b.* All successful essays become the property of the Association. *c.* The medal shall be conferred and honorable mention made of the two other essays considered worthy of this distinction, at a general meeting of the Association. *d.* The competition is to be confined to those who at the time of entering the competition, as well as at the time of conferring the medal, shall be members of the American Medical Association. *e.* The competition for the medal will be closed three months before the next annual meeting of the American Medical Association, and no essays will be received after March 1, 1902.

Communications may be addressed to any member of the committee, consisting of the following: Dr. Herbert L. Burrell, 22 Newberry Street, Boston, Mass.; Dr. Edward Martin, 415 S. 15th Street, Philadelphia, Pa.; Dr. Charles H. Mayo, Rochester, Minn.

